# Variations in the body surface temperature of sows during the post weaning period and its relation to subsequent reproductive performance

**DOI:** 10.5713/ajas.19.0576

**Published:** 2019-08-26

**Authors:** Ruey-Chee Weng

**Affiliations:** 1 Department of Animal Science, National Pingtung University of Science and Technology, Neipu Pingtung 91201, Taiwan

**Keywords:** Sow, Estrus, Weaning, Infrared Technology, Reproduction

## Abstract

**Objective:**

A study was made investigate factors affecting body surface temperature changes after weaning in sows, whether these can be used to aid detection of natural estrus and how they relate to subsequent reproductive performance.

**Methods:**

A total of 132 sows were selected during summer from a breeding farm, with mean parity of 3.6±2.3 and 28.5±0.9 days lactation length. Four daily measurements (6:00, 8:00, 16:00, and 18:00) of vulva (VST), udder (UST), ear base and central back skin temperatures for individual sows were taken by an infrared thermometer, continuing up to 8 days post weaning.

**Results:**

The VST obtained from sows showing estrus at 4 days post-weaning (4DPW), 5DPW, and 6DPW showed a peak at the fourth day post-weaning, but then started to decrease. The VST of sows not detected in standing heat (NDPW) remained at a lower level during the experiment, but UST was increased soon after weaning. The VST-UST temperature differences during daytime of sows that were showing behavioural standing heat on 4DPW, 5DPW, 6DPW, and 7DPW were 0.46°C±0.123°C, 0.71°C±0.124°C, 0.66°C ±0.171°C, and 0.58°C±0.223°C, respectively. The NDPW sows had the highest UST observed, but also the lowest VST so that a more negative value of temperature difference (−0.31°C) was seen during first few days post-weaning. A total of 119 sows were observed to show standing heat and were bred. The later the estrus, the smaller the litter size (p = 0.005).

**Conclusion:**

Sows which did not show behavior indicative of stable standing heat after weaning had a VST which remained at a lower level, but the UST increased soon after weaning. Therefore, for sow heat detection under field conditions, the changes of VST and UST and difference between the two should be considered together to increase the accuracy of detection.

## INTRODUCTION

Recent developments in artificial intelligence technologies have enabled them to continuously monitor biological and environmental information using less labor-intensive approaches for animal production systems. In particular, the use of fully automated data recording or phenotyping platforms based on digital images, sensors, sounds, unmanned systems, and real-time noninvasive computer vision are gaining momentum and have great potential to enhance product quality, management practice, welfare, sustainable development, and animal health [1,2]. The new concept of Precision Livestock Farming may establish the use of such systems by making them easier for management and facilitating prevention of poor welfare by identifying early stages of disease and stressful situations.

Based on animal welfare and effective management considerations, human observation of animal behavior and physiological responses is becoming more critical and is increasingly aided by technological innovation. In reproductive management, artificial insemination, estrus synchronization, and ultrasonography for pregnancy diagnosis are now widely used. However, determining the optimal time to inseminate still relies on a relatively subjective evaluation of behavioral and physical signs of the sow. Although the best fertility results are achieved when artificial insemination is performed during the 24 hours before ovulation [3,4], it is still impossible to reliably predict the onset of estrus in a sow. The development of a technique that could accurately predict estrus timing under field conditions would be of significant importance, since this would allow a more accurate insemination time, and thus an increase in the farrowing rate.

Several studies have tested the applicability of ultrasonography to detect or predict the occurrence of ovulation, but the protocols are time-consuming, require well-trained staff [5,6] and are not able to predict ovulation in advance [7]. Other authors have found a relationship between the electrical resistance of the vaginal mucus and the day of estrus [8,9], but the results show a significant variation among animals and between different measurement locations in the vagina [9,10]. More recently, infrared thermography was used to detect changes in vulvar skin temperatures (VST) of sows during the pre-ovulatory period [11,12]. A significant decrease in VST before ovulation was reported, but it remained unclear whether the temperature peaks observed occurred exclusively in the vulva region or were an associated response to a general increase in body temperature. It has been shown that estrogen administration can induce an increase in vaginal blood flow, measured through an increase in vaginal thermal conductance, in dairy cattle [13]. The increased regional blood flow linked to rising plasma estrogens is reflected by vulva reddening and swelling, which have been described as typical signs of estrus in the sow [14, 15]. However, these research findings mainly focused on VST and some of the experimental animals were hormonally induced into heat.

The aim of this study was to investigate factors affecting body surface temperature changes after weaning in sows, whether these can be used to aid detection of natural sow estrus and determine the most appropriate breeding time, and how they relate toon the subsequent reproductive performance.

## MATERIALS AND METHODS

### Animal care

The experiments were carried out at a legal commercial breeding pig farm located in the north-east of Taiwan, with a capacity of 800 pure breed sows and operated in accordance with the animal protection regulations of the farm management. The experimental design was comparing body surface temperature changes after weaning in sows by using a non-contact body temperature measurement. All procedures were in accordance with the Animal Production Act for Care and Use of Agricultural Animals in Research (Amended Date: 2018-12-26) and approved by the Council of Agriculture, Taiwan, ROC.

### Animals and housing

The experimental group comprised a total of 132 sows, with 50 Duroc, 50 Landrace, and 32 Large White sows studied during the summer season. The sow replacement rate at the farm was 25%, with a mean parity in the experimental group of 3.6±2.3 and lactation length of 28.5±0.9 days. In the previous lactation stage, each sow was housed in a farrowing crate inside a farrowing pen, which was 1.80 meters wide and 2.40 meters long. Maximum length of the crate was 2.25 meters. Feed allowance was individually adjusted for each sow after parturition, taking into account litter size and body condition of the sow. Piglets were able to move about freely in the pen. The piglet creep, on the side of the pen closest to the wall, could be heated with a heater hanging on the lid of the creep nest. The sow and piglet performance data were collected for both on-farm and central performance test programs. No cross-fostering was carried out, but a nurse sow was used in case of emergency and weakness. The weaning process was simply to move the sow to a group pen without any hormonal treatment or feed additive in the diet.

Measurements of body temperature by infrared methods took place in the grouping pen, where sows were housed immediately after weaning. In this pen, sows were kept on partially slatted concert floors without straw bedding, had *ad libitum* access to fresh water from nipple drinkers hanging on the gate located in the front of the pen, and received manual feeding in a trough twice a day at 7:00 and 17:00. The feed was a corn-soy commercial lactation diet containing 18% crude protein and 3,400 Kcal DE per kilogram. The observations were conducted over 8 consecutive days post weaning for each sow. The room temperatures during the experiment ranged from 27.4°C to 30.1°C, with an average of 28.84°C± 2.37°C.

### The infrared thermometer

An infrared thermometer is a noninvasive and safe technique that measures the temperature of a surface, based on its emission of infrared radiation. Soerensen et al [16] determined that the emissivity values for a sow’s ear base, udder, and shoulder (hairy) were 0.978±0.006, 0.975±0.006, and 0.946±0.006, respectively. The results of their study confirm that it is valid to use the human skin emissivity value of 0.98 for infrared skin measurements on sows. However, when studying hairy skin areas or skin with no blood perfusion, the emissivity value is lower. In our experiment, an infrared thermometer (FUNET EM-350B, E-News Hardward Co., Ltd. Taipei, Taiwan) was used to measure the target points from a distance of 30 to 50 cm. This meter has a resolution limit of 0.1°C/0.1°F and a fixed emissivity of 0.95. The non-contact infrared thermometer has a reaction time of 0.5 seconds, so it takes less than one minute for each pig to be measured at four target points.

### Body surface temperature measurement

This farm implemented a weekly weaning operation so that 40 to 48 sows were weaned every week. Soon after weaning, 3 to 4 sows were grouped in a 3 m×4 m pen for heat detection. There were two experimentalists simultaneously recording data for sows in eight grouping pens, therefore, data from 24 to 32 sows can be collected weekly. The body temperatures of the sows were measured four times daily at 6:00, 8:00, 16:00, and 18:00, avoiding feeding times of 7:00 and 17:00. Infrared temperature was measured in the following four body surface target sites: the vulva, the udder (upper part of the anterior of the two mammary glands) the ear base (on the back of the ear) and the back (P2 location, at the level of the last rib and 6.5 cm from the midline). These target sites were selected on the basis of previous research results [11,12,17,18]. Some temperature data were excluded from the study because of the time at which sows were moved from the lactation house to the group pen on the first day post weaning.

### Detection of estrus and experimental group definition

Starting from the first day post weaning, estrus was monitored twice daily (6:30 and 17:30) by using rotated mature boars which were moved into the front aisle of the pen. Estrus definition was based on the recognition of the typical signs of standing reflex and ear pricking after back pressure by a skilled person. The sows detected in the morning or in the afternoon were recorded as starting estrus at the same day, and placed in the same treatment group for statistical analysis. This gave 5 groups: those detected on the 4th-day-postweaning (4DPW), the 5th-day-postweaning (5DPW), the 6th-day-postweaning (6DPW), the 7th-day-postweaning (7DPW) and those which had no heat detected within 8 days post weaning (NDPW). Because the temperature variables of the first standing heat might be susceptible to the time of day, the temperature variables were also categorized for AM and PM sub-groups of the first observed standing heat. After first detection of standing heat, the sows were served by using artificial insemination twice at a 10 to 12 hours interval. Pregnancy checks by ultrasound (PREG-TONE New Series 6, Renco Corporation, Minneapolis, MN, USA) were done at 21 and 42 days after insemination. The subsequent farrowing rate data were only collected from sows that showed signs of the first standing heat within 8 days post-weaning and were successful bred at this period.

### Statistical analysis

SPSS software [19] was used to compare the characteristics of body surface temperatures at one hour before and after the two daily feedings from day 1 to day 8 post-weaning. Analysis of variance was also used to compare the characteristics of subsequent reproduction according to the day on which sows were detected in standing heat. To determine the effects of breed, previous parity, previous reproduction performance, the day to be detected in standing heat and the time to be detected in standing heat, these factors were placed as main effects in a general linear model with each of the skin temperatures as dependent variable. Four sites temperature measures of a sow were included as a random factor to account for repeated measures. Other variables were created based on the difference between vulva and udder (vulva – udder = VU), vulva and ear base (vulva – ear base = VE) and vulva and central back (vulva – central back = VB). These allowed us to differentiate temperature changes due to a general physiological reaction, rather than a specific vulvar reaction. Where a significant main effect of a factor was determined, a Tukey’s honestly significant difference post hoc test was performed to determine the significance of differences between its individual component means. An α level of 0.05 was considered statistically significant.

## RESULTS

### General conditions

The room temperatures ranged from 27.4°C to 30.1°C, with the highest temperatures recorded at 18:00 (30.1°C±2.3°C) and the lowest at 6:00 (27.4°C±1.2°C). Under summer environmental conditions, there was a gradual increase from day 1 (27.6°C±2.1°C) to day 8 (29.3°C±2.3°C) during the progress of this trial.

### Feeding effect

A significant daily variation pattern in the VST was found for the one hour before and after each of the two daily feedings (p<0.001; Table 1), with the highest temperatures obtained at 8:00 and 18:00 after feeding. Similar daily variation patterns were found for the udder (p<0.001), ear base (p<0.001), and central back area (p<0.001).

### Post weaning interval effect

The vulva skin was measured to have the highest temperature on day 4 post weaning, but the lowest on day 1 and day 8 post-weaning (p<0.001; Table 2). Similar measurement variation patterns were found for ear base skin temperatures (p<0.001). The udder skin temperatures (UST) remained relatively high for the first 4 days post weaning, and then gradually decreased until day 8 post-weaning (p<0.001). Soon after weaning, sows tended to have the lowest central back skin temperature observed, at 33.36°C, and then started to increase to 34.74°C by day 7 post-weaning (p<0.001).

### Breed, parity, pre-weaning litter sizes effects

There were 50 Landrace, 32 Large White, and 50 Duroc sows measured in this study (Table 3). Duroc sows were measured to have lower UST (p<0.001) and ear base skin (p<0.001) temperatures than the other two breeds, but no significant difference were seen for VST (p = 0.910) temperatures or for central back area (p = 0.971) temperatures. Younger sows of the first and second parity were seen to have higher VST (p = 0.002; Table 4), but no significant difference showed for ear base skin temperature (p = 0.222) or for central back area temperature (p = 0.644). The sows which nursed a smaller litter size in the previous lactation stage had a lower VST (p< 0.002; Table 5), the lowest UST (p<0.001) and the lowest ear base skin temperature (p<0.001) during the weaning to heat interval.

### Different days of observed standing heat effects and interactions

None of the 132 sows observed in this trial were hormonally treated to induce estrus. Table 6 shows the different days on which estrus was observed during the experiment, for which the number of sows in standing heat on 4, 5, 6, 7, and 8 days post weaning were 40, 39, 20, 13, and 7, respectively. The latter 7 sows showed unstable standing heat behavior and 13 sows did not react at all to the boar contact regime; these were grouped together as NDPW sows and were measured to have the highest UST (p<0.001) but the smallest temperature difference between vulva and udder measurements (p< 0.001). In addition, the subsequent farrowing rate for 4DPW, 5DPW, 6DPW, and 7DPW sows were 67.5% (27/40), 82.0% (32/39), 85.0% (17/20), and 69% (9/13), respectively (Tables 6, 7).

**Figure 1:**
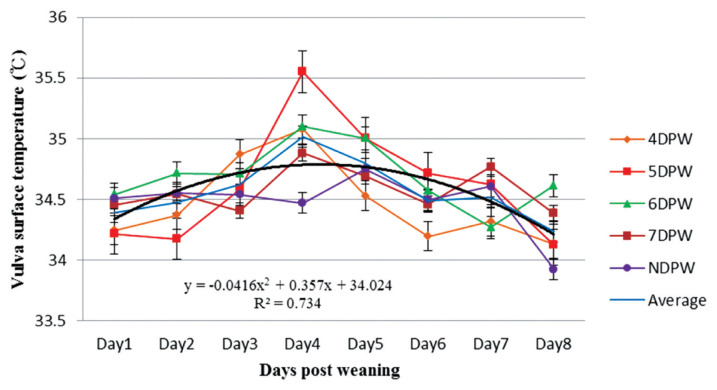
The distribution of sow’s vulva surface temperature (°C) from day 1 to day 8 post-weaning for sows with different days to first show behavioural standing heat. The temperatures of sows were measured four times daily by using an infrared thermometer and following the dynamics continuously up to 8 days post weaning (n = 132). DPW, day post-weaning on which heat was first shown; NDPW, day post-weaning on which not detected in standing heat.

**Figure 2:**
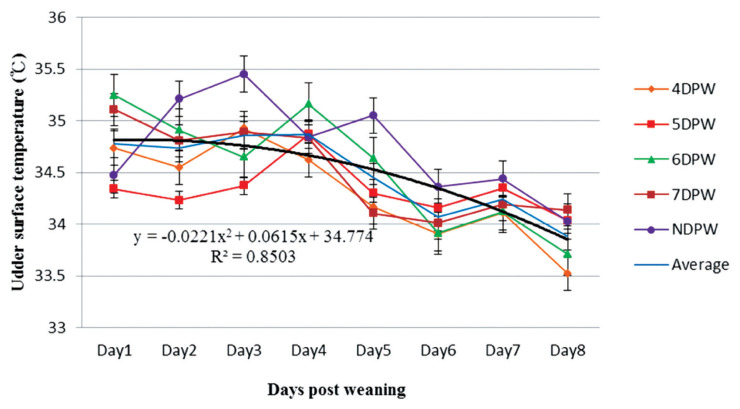
The distribution of sow’s udder surface temperature (°C) from day 1 to day 8 post-weaning for sows with different days to first show behavioural standing heat. The temperatures of sows were measured four times daily by using an infrared thermometer and following the dynamics continuously up to 8 days post weaning (n = 132). DPW, day post-weaning on which heat was first shown; NDPW, day post-weaning on which not detected in standing heat.

**Figure 3:**
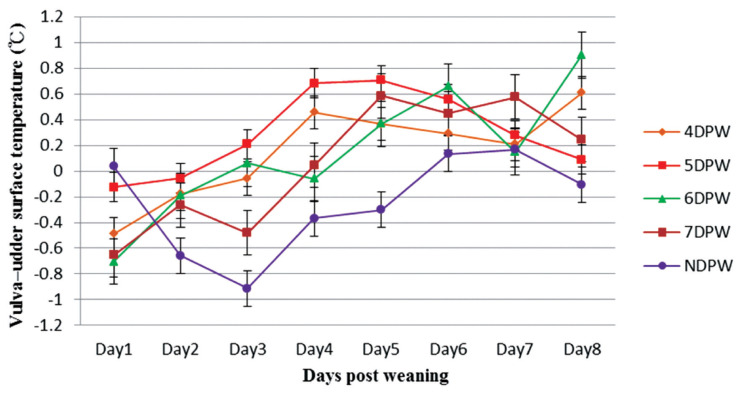
The variation in vulva–udder surface temperature (°C) from day 1 to day 8 post-weaning for sows with different days to first show behavioural standing heat. The vulva–udder temperature differences of sows were compared four times daily and following the dynamics continuously up to 8 days post weaning (n = 132). DPW, day post-weaning on which heat was first shown; NDPW, day post-weaning on which not detected in standing heat.

The analysis of VST obtained from 4DPW, 5DPW, and 6DPW sows showed an increase to the fourth day post-weaning, but then started to decrease (Figure 1). The VST of NDPW sows remained at a lower level during the experiment, but the UST increased soon after weaning (Figure 2). Variation in VU surface temperature for different days when the sows showed behaviors of standing heat sows from day 1 to day 8 post-weaning is illustrated in Figure 3, showing that for each group the V-U temperature increased up until the day of heat then started to drop. The mean temperature differences for 4DPW, 5DPW, 6DPW, and 7DPW sows on the day of standing heat were 0.46°C±0.123°C, 0.71°C±0.124°C, 0.66°C±0.171°C, and 0.58°C±0.223°C, respectively. The NDPW sows had the highest UST observed, but also the lowest VST. Therefore, a more negative contrast value (−0.31°C) was calculated after the first days post-weaning.

The variation in VU surface temperature at that time of first observed standing heat for different time of day subgroups is compared. Both AM and PM subgroups in NDPW had a negative difference value between VST and UST, because there was no significant increase in VST temperature of these two subgroups and UST remained higher for them. Most of the sows in both groups were not observed to show a stable standing estrus. Both AM and PM subgroup in 4DPW, 5DPW, 6DPW, and 7DPW had a positive difference value between VST and UST. However, 4DPW-AM subgroup and 7DPW-PM subgroup had lower VU surface temperature differences. The lower VU figures were associated with a lower farrowing rate in the subsequent reproductive performance. The farrowing rate for 4DPW-AM subgroup and 7DPW-PM subgroup were 43% (6/14) and 43% (3/7), respectively (Table 7). The VU values of the remaining subgroups were relatively high and the farrowing rate was more than 78% (Table 7) in the subsequent reproductive performance.

Measurement of subsequent reproductive performance (Table 7) showed that the later the estrus, the smaller the litter size (p = 0.005). However, the number of live piglets was not significantly different at 5 days post parturition (p = 0.051).

## DISCUSSION

This study was focused on measuring the skin temperatures of sows in different areas and following the dynamics continuously up to 8 days post weaning. The temperature varied in the vulva, udder, ear base and central back regions throughout the day, with lower temperatures obtained in the early morning (6:00) and higher temperatures in the late afternoon (18:00). These findings are in agreement with the results from other studies that monitored the vaginal temperature using internal temperature transmitters [12,20]. These daily fluctuations have been generally defined in literature by means of the circadian rhythm, an endogenous thermal regulation mechanism responsible for a temperature fall during the night period. An effect of feeding time may have been present, with higher temperature after feeding as a result of increased activity and/or heat of digestion. The changes in daily environmental temperature also contributed to the explanation of variations in the highest and lowest skin temperatures recorded at 18:00 PM and 6:00 AM, respectively, which corresponded with the maximum and minimum room temperatures measured at the same periods of the day.

We found the vulva skin was measured to have the highest temperature on day 4 post-weaning for all oestrus groups except NDPW, with the lowest values measured at day 1 and day 8 post-weaning. Similar measurement variation patterns were found for ear base skin temperatures. These findings were in agreement with the results from other studies [11,17]. Simões et al [12] failed to detect the increase in VST and their results were affected by the steady decrease in room temperature throughout the experiment. The same authors also stated that vulvar temperature seems to rise before estrus. In a similar study, a significant increase was reported in the VST as estrus approached and a drop before ovulation (−1.5°C) [11]. Sykes and colleagues [17] tested infrared thermal imaging (IRT) in gilts to differentiate animals in estrus and non-estrus, and reported significantly higher VST in the estrus period. Thus, the IRT technique can probably be used for early detection of the onset of estrus, especially in animals with silent estrus. Ear base temperature seems to rise approximately 1°C before standing estrus, while vaginal temperature seems to drop approximately 0.5°C [18]. However, these research findings mainly focus on VST and some of the experimental animals were hormonally induced to come into heat. A better synthesis of results from studies under different conditions is needed before results can be fully applied in field conditions.

In this study, the USTs remained relatively high for the first 4 days post weaning, and then gradually decreased to day 8 post-weaning (p<0.001). This might be explained by the stress from weaning process. Sows experience stress from piglet removal and changes of housing environment, as well as the transition of the mammary tissue into the dry period, all within day 4 to 5. A reduction in metabolic activity in the udder should occur after weaning as milk synthesis ceases. However, the UST remained higher after weaning for NDPW sows, possibly reflecting subclinical udder infection and inflammation in these sows. There are no previous research results concerning the relationship between diagnosis of the reproductive state and UST.

In this study, temperatures at different sites were measured to compare among breeds, parities and sows with different levels of productivity. Duroc sows were measured to have lower USTs than other two breeds, although no significant differences were seen for VSTs. Sows which nursed a larger litter size in previous lactation stage had a higher VST and the highest UST. It is therefore necessary to take account of such effects if a threshold for VST is to be developed for estrus detection.

A more reliable indicator of the time of estrus than VST alone seemed to be the VU temperature difference. Each group showed a peak value at the time of behavioural estrus, suggesting possible use of this measure in automated estrus detection. In contrast, the analysis of VE and VB temperature differences did not show a regular relationship with the different duration from weaning to estrus.

As has been observed previously, the later the estrus the lower the subsequent litter size. However, the number of live piglets was not significantly different at 5 days post parturition. This might reflect greater neonatal mortality in large litters in this experiment the influence of external factors was controlled as much as possible, but procedures took place within the scope of general commercial management. The results of the data analysis are similar to those of the previous controlled experiments. For example, sows with prolonged weaning to first mating interval had a lower farrowing rate and fewer piglets born alive than those with an interval of 3 to 6 days [21,22]. A prolonged weaning to first mating interval is related to a short duration of estrus and to a shorter interval between onset of estrus and ovulation [23,24]. One consequence of this is an increased risk of inseminating at a suboptimal time, which can be a major cause of low farrowing rate and fewer piglets born alive.

## CONCLUSION

Use of an infrared thermometer does not require any contact and is therefore a completely non-invasive technique that allows measurements on subjects difficult to reach or to approach, or moving subjects. In sow heat detection; this technique could be used for mass screening aimed at the early diagnosis of heat, characterized by an increase of VST. However, factors such as metabolic activity of the mammary tissue into the dry period, variation in behavior changes, level of physical activity, fluctuations in the environmental temperature, management level and the animal’s body condition can cause a considerable variation in skin temperature, which may limit the applicability of this technology.

The difference between vulva and udder temperature seems to give a more reliable indication of estrus. The temperature of the vulva skin must reach 0.5 degrees higher than the temperature of the udder skin if the sow will show a stable estrus behavior at 4 to 7 days post weaning. Therefore, for sow heat detection under field conditions, the temperature difference between the vulva and udder should be considered to increase the accuracy of detection.

## Figures and Tables

**Table 1:** The main effect of observing time on temperature measurements (°C) of the body surface of sows

Items	Time (n = 1,056)1)				SEM	p-value

	06:00	08:00	16:00	18:00		
Body surface						
Vulva	34.24±0.054^c^	34.79±0.054^a^	34.54±0.050^b^	34.79±0.051^a^	0.039	<0.001
Udder	34.32±0.056^b^	34.54±0.056^a^	34.33±0.051^b^	34.61±0.053^a^	0.042	<0.001
Ear	33.41±0.065^c^	34.49±0.065^a^	33.89±0.060^b^	34.59±0.062^a^	0.058	<0.001
Back	33.57±0.052^c^	34.40±0.052^a^	34.05±0.048^b^	34.39±0.049^a^	0.036	<0.001
Temperature difference between target sites						
VU	−0.07±0.054^b^	0.26±0.054^a^	0.22±0.050^a^	0.19±0.052^a^	0.040	<0.001
VE	0.83±0.055^a^	0.30±0.055^c^	0.66±0.051^b^	0.20±0.053^c^	0.042	<0.001
VB	0.67±0.073^a^	0.40±0.073^c^	0.49±0.067^ab^	0.40±0.070^c^	0.072	0.024

Value shown is the mean±standard deviation from day 1 to day 8 post-weaning.

SEM, standard error of the mean; VU, vulva - udder; VE, vulva - ear base; VB, vulva - central back.

1) n, sample size = 132 sows×8 days.

a–c Means within a row without a common superscript letter differ (p<0.05).

**Table 2:** The main effect of day after weaning on temperature measurements (°C) of the body surface of sows

Items	Post-weaning (day, n = 528)1)								SEM	p-value

	1	2	3	4	5	6	7	8		
Body surface										
Vulva	34.40^d^ ±0.094	34.39^d^ ±0.067	34.66^bc^ ±0.067	35.15^a^ ±0.069	34.83^b^ ±0.068	34.48^cd^ ±0.068	34.48^cd^ ±0.076	34.15^e^ ±0.092	0.029	<0.001
Udder	34.71^a^ ±0.096	34.61^ab^ ±0.068	34.77^a^ ±0.069	34.82^a^ ±0.070	34.40^bc^ ±0.069	34.05^d^ ±0.070	34.21^cd^ ±0.078	33.76^e^ ±0.094	0.031	<0.001
Ear	33.76^d^ ±0.117	33.76^d^ ±0.083	34.25abc ±0.084	34.50^a^ ±0.086	34.33^ab^ ±0.084	34.01^cd^ ±0.085	34.15^bc^ ±0.095	33.81^d^ ±0.114	0.037	<0.001
Back	33.36^f^ ±0.089	33.49^f^ ±0.063	33.82^e^ ±0.064	34.11^d^ ±0.065	34.48^bc^ ±0.064	34.30^cd^ ±0.065	34.74^a^ ±0.072	34.64^ab^ ±0.087	0.027	<0.001
Temperature difference between target sites										
VU	−0.32^b^ ±0.094	−0.23^b^ ±0.067	−0.11^b^ ±0.067	0.33^a^ ±0.069	0.43^a^ ±0.067	0.44^a^ ±0.068	0.27^a^ ±0.076	0.39^a^ ±0.091	0.029	<0.001
VE	0.64a ±0.098	0.63^a^ ±0.070	0.41^ab^ ±0.070	0.66^a^ ±0.072	0.50^ab^ ±0.071	0.47^ab^ ±0.071	0.34^b^ ±0.080	0.34^b^ ±0.096	0.030	0.014
VB	1.05^a^ ±0.125	0.93^a^ ±0.089	0.84^a^ ±0.089	0.99^a^ ±0.091	0.31^b^ ±0.089	0.18^b^ ±0.090	−0.26^c^ ±0.101	−0.46^c^ ±0.121	0.040	<0.001

Value shown is the mean±standard deviation from day 1 to day 8 post-weaning.

SEM, standard error of the mean; VU, vulva - udder; VE, vulva - ear base; VB, vulva - central back.

1) n, sample size = 132 sows×4 measurements per day.

a–d Means within a row without a common superscript letter differ (p<0.05).

**Table 3:** The main effect of sow breed on temperature measurements (°C) of the body surface of sows

Items	Breed Observations	Landrace (n = 50)1)	Large White (n = 32)2)	Duroc (n = 50)3)	SEM	p-value
Body surface						
Vulva		34.61±0.041^W^	34.64±0.057^W^	34.60±0.043^W^	0.030	0.910
Udder		34.56±0.042^W^,^a^	34.47±0.058^W^,^a^	34.30±0.044^X^,^b^	0.032	<0.001
Ear		34.27±0.051^X^,^a^	34.17±0.070^X^,^a^	33.91±0.053^Z^,^b^	0.037	<0.001
Back		34.08±0.040^Y^	34.07±0.055^X^	34.07±0.042^Y^	0.030	0.971
SEM		0.021	0.029	0.022	-	-
p-value		<0.001	<0.001	<0.001	-	-
Temperature difference between target sites						
VU		0.05±0.041^b^	0.17±0.056^ab^	0.30±0.043^a^	0.030	<0.001
VE		0.34±0.042^b^	0.46±0.058^b^	0.70±0.044^a^	0.029	<0.001
VB		0.47±0.055	0.51±0.076	0.48±0.057	0.042	0.922

Value shown is the mean±standard deviation from day 1 to day 8 post-weaning.

SEM, standard error of the mean; VU, vulva - udder; VE, vulva - ear base; VB, vulva - central back.

1) Sample size = 50 Landrace sows×4 measurements per day×8 days.

2) Sample size = 32 Large White sows×4 measurements per day×8 days.

3) Sample size = 50 Duroc sows×4 measurements per day×8 days.

a,b Means within a row without a common superscript letter differ (p<0.05).

W–Z Means within a column without a common superscript letter differ (p<0.05).

**Table 4:** The main effect of parity on temperature measurements (°C) of the body surface of sows

Items	Parity Observations	1 to 2 d (n = 50)1)	3 to 4 d (n = 39)2)	5 to 6 d (n = 22)3)	≥7 d (n = 21)4)	SEM	p-value
Body surface							
Vulva		34.74±0.042^W^,^a^	34.59±0.048^W^,^ab^	34.45±0.064^W^,^b^	34.45±0.069^W^,^b^	0.031	0.002
Udder		34.46±0.044^X^,^a^	34.51±0.049^W^,^a^	34.23±0.066^WX^,^b^	34.50±0.071^W^,^a^	0.033	0.017
Ear		34.09±0.052^Y^	34.14±0.059^X^	33.99±0.079^Y^	34.25±0.085^WX^	0.038	0.222
Back		34.04±0.041^Y^	34.11±0.047^X^	34.04±0.063^XY^	34.12±0.067^X^	0.031	0.644
SEM		0.022	0.025	0.034	0.035	-	-
p-value		<0.001	<0.001	<0.001	<0.001	-	-
Temperature difference between target sites							
VU		0.28±0.042^a^	0.08±0.048^b^	0.25±0.064^a^	−0.16±0.068^b^	0.031	0.001
VE		0.65±0.044^a^	0.45±0.049^b^	0.49±0.066^ab^	0.23±0.071^c^	0.030	<0.001
VB		0.63±0.057^a^	0.42±0.064^ab^	0.40±0.086^ab^	0.32±0.092^b^	0.044	0.025

Value shown is the mean±standard deviation from day 1 to day 8 post-weaning.

SEM, standard error of the mean; VU, vulva - udder; VE, vulva - ear base; VB, vulva - central back.

1) Sample size = 50 sows×4 measurements per day×8 days.

2) Sample size = 39 sows×4 measurements per day×8 days.

3) Sample size = 22 sows×4 measurements per day×8 days.

4) Sample size = 21 sows×4 measurements per day×8 days.

a–c Means within a row without a common superscript letter differ (p<0.05).

W–Y Means within a column without a common superscript letter differ (p<0.05).

**Table 5:** The main effect of pre-weaning litter size on temperature measurements (°C) of the body surface of sows

Items	Litter size Observations	≤6 d (n = 23)1)	7 to 8 d (n = 36)2)	9 to 10 d (n = 39)3)	≥11 d (n = 34)4)	SEM	p-value
Body surface							
Vulva		34.39±0.063^W^,^b^	34.73±0.050^W^,^a^	34.61±0.048^W^,^a^	34.64±0.052^W^,^a^	0.029	<0.002
Udder		33.98±0.065^X^,^c^	34.52±0.051^X^,^b^	34.42±0.049^W^,^b^	34.71±0.053^W^,^a^	0.030	<0.001
Ear		33.67±0.078^Y^,^c^	34.35±0.061^Y^,^a^	34.03±0.059^X^,^b^	34.26±0.064^X^,^a^	0.036	<0.001
Back		34.07±0.062^X^	34.15±0.049^Z^	34.01±0.047^X^	34.08±0.051^X^	0.030	0.304
SEM		0.036	0.025	0.025	0.027	-	-
p-value		<0.001	<0.001	<0.001	<0.001	-	-
Temperature difference between target sites							
VU		0.41±0.063^a^	0.21±0.050^b^	0.19±0.048^b^	−0.07±0.052^c^	0.029	<0.001
VE		0.73±0.065^a^	0.38±0.051^b^	0.58±0.049^a^	0.37±0.054^b^	0.029	<0.001
VB		0.25±0.085	0.51±0.067	0.55±0.064	0.53±0.070	0.041	0.081

Value shown is the mean±standard deviation from day 1 to day 8 post-weaning.

SEM, standard error of the mean; VU, vulva - udder; VE, vulva - ear base; VB, vulva - central back.

1) Sample size = 23 sows×4 measurements per day×8 days.

2) Sample size = 36 sows×4 measurements per day×8 days.

3) Sample size = 39 sows×4 measurements per day×8 days.

4) Sample size = 34 sows×4 measurements per day×8 days.

a–c Means within a row without a common superscript letter differ (p<0.05).

W–Z Means within a column without a common superscript letter differ (p<0.05).

**Table 6:** Temperature measurements (°C) of the body surface of sows analyzed for different days of observed standing heat

Items	Observations2) Subgroups3)	4DPW1) (n = 40)5) (14:26)	5DPW (n = 39)6) (16:23)	6DPW (n = 20)7) (12:8)	7DPW (n = 13)8) (6:7)	NDPW1) (n = 204))9) (5:15)	SEM	p-value
Body surface								
Vulva		34.50±0.047^W^,^b^	34.68±0.047^W^,^ab^	34.75±0.069^W^,^a^	34.62±0.076^W^,^ab^	34.54±0.078^WX^,^ab^	0.032	0.041
Udder		34.31±0.048^X^,^c^	34.34±0.048^X^,^bc^	34.56±0.071^W^,^b^	34.53±0.078^W^,^b^	34.83±0.079^W^,^a^	0.033	<0.001
Ear		33.81±0.058^Y^,^b^	34.23±0.058^XY^,^a^	34.28±0.085^X^,^a^	34.21±0.094^X^,^a^	34.31±0.095^X^,^a^	0.039	<0.001
Back		33.81±0.045^Y^,^c^	34.10±0.046^Y^,^b^	34.26±0.067^X^,^ab^	34.32±0.074^WX^,^a^	34.27±0.075^X^,^ab^	0.032	<0.001
SEM		0.024	0.024	0.036	0.041	0.041	-	-
p-value		<0.001	<0.001	<0.001	0.001	<0.001	-	-
Temperature difference between target sites								
VU		0.19±0.046^ab^	0.34±0.047^a^	0.19±0.069^ab^	0.09±0.075^b^	−0.28±0.077^c^	0.031	<0.001
VE		0.70±0.048^a^	0.45±0.049^b^	0.47±0.071^b^	0.41±0.078^bc^	0.24±0.080^c^	0.031	<0.001
VB		0.68±0.063^a^	0.48±0.063^ab^	0.44±0.093^ab^	0.27±0.102^b^	0.22±0.104^b^	0.045	0.003

Value shown is the mean±standard deviation from day 1 to day 8 post-weaning.

SEM, standard error of the mean; VU, vulva - udder; VE, vulva - ear base; VB, vulva - central back.

1) DPW, day post-weaning on which heat was first shown; NDPW, day post-weaning on which not detected in standing heat.

2) Number of sows observed standing heat.

3) Number of sows in AM and PM subgroups (AM:PM).

4) Number of sows observed non-standing heat.

5) Sample size = 40 sows×4 measurements per day×8 days.

6) Sample size = 39 sows×4 measurements per day×8 days.

7) Sample size = 20 sows×4 measurements per day×8 days.

8) Sample size = 13 sows×4 measurements per day×8 days.

9) Sample size = 20 sows×4 measurements per day×8 days.

a–c Means within a row without a common superscript letter differ (p<0.05).

W–Y Means within a column without a common superscript letter differ (p<0.05).

**Table 7:** The subsequent reproductive performance of sows observed to show standing heat at different days post-weaning

Items	4DPW 271) (6:21)2) (43:81)3)	5DPW 32 (14:18) (88:78)	6DPW 17 (10:7) (83:88)	7DPW 9 (6:3) (100:43)	SEM	p-value
Litter size at birth, pigs	10.05±0.20^a^	9.38±0.17^a^	9.64±0.25a	8.54±0.42^b^	0.555	0.005
Litter weight at birth, kg	12.39±0.17^ab^	11.94±0.14^b^	13.04±0.21^a^	12.27±0.36^ab^	0.919	0.040
Live piglets at 5-day-old, pigs	8.19±0.26	7.49±0.22	8.09±0.32	7.63±0.54	0.392	0.051

Value shown is the mean±standard deviation.

DPW, day post-weaning; SEM, standard error of the mean.

1) Number of sows observed with successful parturition.

2) Number of successful parturition sows in AM and PM subgroups (AM:PM).

3) Farrowing rate of sows in AM and PM subgroups (AM %:PM %).

a,b Means within a row without a common superscript letter differ (p<0.05).
